# Navigating rice seedling cold resilience: QTL mapping in two inbred line populations and the search for genes

**DOI:** 10.3389/fpls.2023.1303651

**Published:** 2023-12-14

**Authors:** Michael R. Schläppi, Avery R. Jessel, Aaron K. Jackson, Huy Phan, Melissa H. Jia, Jeremy D. Edwards, Georgia C. Eizenga

**Affiliations:** ^1^Department of Biological Sciences, Marquette University, Milwaukee, WI, United States; ^2^Dale Bumpers National Rice Research Center, U.S. Department of Agriculture, Agricultural Research Service (USDA-ARS), Stuttgart, AR, United States

**Keywords:** Asian rice, chilling tolerance, genome-wide association study, heading date, *Indica* rice, *Japonica* rice, *Oryza sativa*, QTL mapping

## Abstract

Due to global climate change resulting in extreme temperature fluctuations, it becomes increasingly necessary to explore the natural genetic variation in model crops such as rice to facilitate the breeding of climate-resilient cultivars. To uncover genomic regions in rice involved in managing cold stress tolerance responses and to identify associated cold tolerance genes, two inbred line populations developed from crosses between cold-tolerant and cold-sensitive parents were used for quantitative trait locus (QTL) mapping of two traits: degree of membrane damage after 1 week of cold exposure quantified as percent electrolyte leakage (EL) and percent low-temperature seedling survivability (LTSS) after 1 week of recovery growth. This revealed four EL QTL and 12 LTSS QTL, all overlapping with larger QTL regions previously uncovered by genome-wide association study (GWAS) mapping approaches. Within the QTL regions, 25 cold-tolerant candidate genes were identified based on genomic differences between the cold-tolerant and cold-sensitive parents. Of those genes, 20% coded for receptor-like kinases potentially involved in signal transduction of cold tolerance responses; 16% coded for transcription factors or factors potentially involved in regulating cold tolerance response effector genes; and 64% coded for protein chaperons or enzymes potentially serving as cold tolerance effector proteins. Most of the 25 genes were cold temperature regulated and had deleterious nucleotide variants in the cold-sensitive parent, which might contribute to its cold-sensitive phenotype.

## Introduction

Rice is one of the most important crops and, due to its subtropical origin, is generally sensitive to even short exposures to cold stress at all stages of development (da Cruz et al., 2013; Li et al., 2022). Because of global climate change, recent weather extremes, such as colder winters affecting early spring rice planting, have become increasingly common (Johnson et al., 2018). Moreover, to either expand rice cultivation into higher latitudes or altitudes where air temperatures can fluctuate widely during the early growing season, it is important to elucidate rice cold stress tolerance response mechanisms that allow young rice seedlings to survive several days of exposure to chilling temperatures and resume growth and development during recovery from stress. Interestingly, because of the way rice was domesticated and spread by humans, this generally cold-sensitive crop has a wide range of temperature adaptation that is structured into subpopulations, and variation for cold tolerance within subpopulations may act through very different mechanisms (Li et al., 2022). These mechanisms can be studied genetically because there is considerable natural variation in cultivated self-pollinating plants such as rice. Previous studies by us and others showed the *Japonica* varietal group (VG), composed of *temperate japonica* (TEJ) and *tropical japonica* (TRJ) accessions, is considerably more cold tolerant than the *Indica* VG, composed of *aus* (AUS) and *indica* (IND) accessions, which allowed us to map various cold tolerance trait QTL using genome-wide association study (GWAS) mapping approaches (Schläppi et al., 2017; Shakiba et al., 2017; Shimoyama et al., 2020; Phan and Schläppi, 2021; reviewed in Li et al., 2022). Since many of these QTL cover relatively large genomic regions containing many genes, a recurrent challenge is to identify cold tolerance candidate genes and ultimately the causal genes and their alleles associated with those QTL.

The purpose of this study was to fine-map cold tolerance trait QTL using bi-parental mapping populations to identify smaller genomic regions that overlap with the larger QTL regions we previously identified through GWAS, thus allowing us to narrow down cold tolerance candidate genes within those smaller regions. Based on our previous results, we selected two robustly cold-tolerant and cold-sensitive parents to generate recombinant inbred lines (RILs) and backcross inbred lines (BILs). The two populations were used to map agricultural traits as “quality control” to assess whether known heading date and plant height QTL and the associated genes could be uncovered. Young RIL and BIL seedlings were then exposed for 1 week to a chilling temperature of 10°C, after which the degree of membrane damage in the leaves of exposed plants was measured, while seedlings’ survival after 1 week of recovery growth was determined. Our results validated the two mapping populations suitable for QTL mapping, and 16 cold tolerance trait QTL were uncovered, all of which overlapped with at least one of the QTL we found by GWAS mapping. The genomic regions delineated by those QTL contained at least 25 candidate genes with polymorphisms between the cold-tolerant and cold-sensitive parents, and their potential contributions to rice cold tolerance are discussed.

## Materials and methods

### Rice materials and inbred line production

The three accessions used to generate inbred lines are part of the rice mini-core collection (RMC). Seeds were obtained from the Genetic Stocks-*Oryza* (GSOR) collection located at the USDA-ARS Dale Bumpers National Rice Research Center (DBNRRC; Stuttgart, Arkansas, USA; https://www.ars.usda.gov/GSOR).

To generate recombinant inbred lines (RILs), the cold-tolerant *temperate japonica* accession Krasnodarskij 3352 (GSOR311787; originally from the Krasnodar region of Western Russia) was crossed as female with the cold-sensitive *aus* accession Carolino 164 (GSRO311654; originally from the Chad region of Africa) to generate F_1_ seeds. The F_2_ seed produced from 22 F_1_ plants was advanced by single seed descent to the F_8_ generation, with 12 RILs only advanced to the F_7_, for a final population of 140 RILs. Leaf tissue was collected from the 140 F_7:8_ RILs and the F_8:9_ seed from 134 (or fewer, if limited seed) of these RILs was used to grow the seedlings for phenotyping.

To generate backcross inbred lines (BILs), the cold-tolerant *temperate japonica* accession WIR 911 (GSOR311685; originally from the Primorsky Krai region of Eastern Russia) was crossed as female with Carolino 164, and the resulting F_1_ was backcrossed as female with Carolino 164, producing 93 BC_1_F_1_ progeny that were advanced by single seed descent to the BC_1_F_5_ generation, with three BILs only advanced to the BC_1_F_4_. Leaf tissue from these 93 BC_1_F_4:5_ BILs was used for genotyping, and BC_1_F_5:6_ seed from 92 (or fewer, if limited seed) of these BILs was used to grow the seedlings for phenotyping.

### Germination and standard growth conditions

For phenotyping, seeds of the three parents, F_8:9_ RILs and BC_1_F_5:6_ BILs were germinated in the dark for 2 days at 37°C in deionized water containing 0.1% bleach to prevent bacterial contamination. Germinating seeds were transferred into PCR strips, placed into pipette tip boxes, and grown hydroponically in deionized water for 10 days in a growth chamber under 12-h light (approximately 150 µE photon flux)/12-h dark and 28°C day/25°C night cycles. To provide nutrients, on day 10, the water was replaced with ¼ Murashige–Skoog basal salt liquid medium. Each line was represented by up to eight plants per box in quadruplicates for up to 32 plants (four boxes of eight plants) per experiment. The four boxes were randomly arranged within the growth chamber. Each box contained 11 strips of RILs or BILs and one strip with the parents as controls. For the RILs, there were four Carolino 164 seedlings, the cold-sensitive control, and four Krasnodarskij 3352 seedlings, the cold-tolerant control. For the BILs, the cold-tolerant parent, WIR 911 was used as the control.

### Chilling stress treatment

The four boxes containing 2-week-old seedlings at the 2-leaf stage were placed at random positions within a growth chamber set at a constant 10°C ± 1°C temperature and incubated for 7 days (12-h light/12-h dark cycles). Watering was done every other day. At the end of the treatment, approximately 1 cm of the middle segments of the second leaf for three seedlings per line was collected for electrolyte leakage (EL) assays, and the boxes were returned to standard growth conditions for a recovery period of 7 days after which low-temperature seedling survivability (LTSS) was recorded (details below).

### Phenotyping of BIL and RIL populations

Agronomic and cold tolerance trait data for the RIL population are summarized in **Supplementary Table S1**. Data for the BIL population are summarized in **Supplementary Table S2**.

### Heading date

Seeds of the F_8:9_ RIL and BC_1_F_5:6_ BILs were germinated and grown under standard growth conditions to generate 14-day-old seedlings. During May 2021, three healthy seedlings per line were transplanted in “hills” into 1.22-m × 1.22-m raised bed paddies on a rooftop at Marquette University in Milwaukee, Wisconsin, 100 lines per paddy (10 rows × 10 columns; 10 cm ± 1 cm distance between seedling “hills”). The heading date was recorded as the number of days from germination to panicle emergence.

### Plant height

The height of the main shoot of each RIL or BIL was measured in cm from the soil level to the tip of the mature panicle.

### Electrolyte leakage

At the end of the 7-day 10°C stress period, approximately 1 cm of the middle section of the second leaf from three individual seedlings per RIL, BIL, or control per box was collected and cut into three equally sized segments. The pieces were washed in deionized water and transferred into three different screw-cap glass tubes filled with 5 mL of deionized water (conductivity ≤ 2 µS/cm), then shaken at 200 rpm for 60 min at room temperature to release cellular electrolytes from low-temperature damaged tissues. The initial conductivity of the three replicates per box (a total of 12 replicates across the four randomly distributed boxes) was measured by taking out 120 µL of the solution and adding it into the well of a hand-held LAQUAtwin B-771 conductivity meter (Horiba Scientific, Japan). Leaf samples were boiled for 20 min after the initial reading to release total cellular electrolytes. Samples were shaken again at 200 rpm for 30 min after cooling to room temperature, and the final conductivity reading was done. The mean percent EL was determined as [(initial conductivity reading)/(final conductivity reading)] × 100.

### Low-temperature seedling survivability

At the end of the 7-day 10°C stress period, seedlings were allowed to recover at standard growth conditions for one week after which seedling survival was determined by visual inspection. Seedlings that were mostly green and stiff were scored as alive while seedlings that were mostly wilted and/or bleached and soft were scored as dead. The mean percent survivability was calculated as [(number of seedlings scored as alive)/(total number of stressed plants)] × 100.

### Statistical analysis of traits

QTL analysis prefers phenotype values for every (RIL or BIL) line in the mapping population. Line means were calculated for the heading date and plant height traits. For the EL and percent LTSS traits, these values were calculated from a linear mixed model (LMM) to obtain the Best Linear Unbiased Predictions (BLUPs) for each line. The LMM to calculate BLUPs for EL and LTSS traits used an augmented design with a fixed effect variable (group) indicating whether the line is a control or RIL (or BIL), a random effect variable for the line nested in the group variable, and random effect variables for the set (growth chamber experiment date) and box. In addition to treating LTSS as a percent value, LTSS was used in a survival analysis with a binomial generalized mixed model (GLMM) and logit link function to predict the probability of survival under cold treatment for each line on the log-odds scale, because percent EL and LTSS data were not normally distributed (**Supplementary Figures S1C, D**). The binomial GLMM used the same fixed and random variables as the LMM used for EL and percent LTSS.

### Genotyping of recombinant inbred lines and QTL mapping

Leaf tissue was harvested from the parents and an individual representative F_7:8_ RIL and BC_1_F_4:5_ plant and lyophilized. Genotyping was done using the Cornell-IR LD Rice Array (C7AIR) containing 7,098 SNP markers (Morales et al., 2020) that was commercially available as an Illumina Infinium array. For DNA extraction and genotyping, lyophilized leaf tissue was sent to Eurofins Diagnostics, Inc. (www.eurofinsgenomics.eu/en/genotyping-gene-expression/service-platforms/illumina-arrayplatforms/).

The IciMapping software (Integrated Software for Building Genetic Linkage Maps and Mapping Quantitative Trait Loci; Meng et al., 2015), version V4.2 (released 12 July 2019) was used to generate a genetic linkage map and to map quantitative trait loci (QTL) via Inclusive Composite Interval Mapping (ICIM). Runs on individual traits with 1,000 permutations (step = 0.5, pin = 0.005, perms 1,000, with significance at 0.05 and 0.1) were done to find LOD cutoffs for each trait, according to the user’s manual (https://isbreedingen.caas.cn/software/qtllcimapping/294607.htm).

### GWAS analysis of the RDP1 and RMC

An earlier GWAS study by Shimoyama et al. (2020) phenotyped 354 accessions included in the Rice Diversity Panel 1 (RDP1) for EL and LTSS. Taking advantage of the increased number of SNPs available through the imputation of the RDP1 genotypes (Wang et al., 2018), the phenotypic data from Shimoyama et al. (2020) was rerun with the imputed genotypes using the mixed linear model in Tassel V 5.0 (Bradbury et al., 2007). The traits examined were EL8, EL10, EL12, LTSS8, LTSS10, and LTSS12, with the numbers 8, 10, and 12 representing the temperature at which the plants were grown, 8°C, 10°C, and 12°C, respectively. To more effectively capture the genetic diversity, seven panels based on subpopulation(s) were used. All panels were filtered to exclude heterozygous markers and markers with a minor allele frequency of less than 5%. The first panel, “353,” was composed of 353 accessions with 2,721,379 SNPs and contained all five rice subpopulations: *indica* (IND), *aus* (AUS), *aromatic* (ARO), *tropical japonica* (TRJ), *temperate japonica* (TEJ), and the admixtures. The subpopulation panels included IND (72 accessions) with 1,637,548 SNPs, AUS (51 accessions) with 1,675,068 SNPs, TRJ (89 accessions) with 921,375 SNPs, and TEJ (86 accessions) with 569,119 SNPs. The ARO subpopulation contained only a limited number of accessions and was included with the 353 set but not as its own panel. The *Indica* VG panel, INDAUS, included both IND and AUS (129 accessions) with 2,389,503 SNPs, and the *Japonica* VG panel, JAP, included TRJ and TEJ (204 accessions) with 1,090,261 SNPs. GWAS run conditions as well as PCA and kinship generation were the same, as summarized in Eizenga et al. (2023).

Schläppi et al. (2017) conducted GWAS mapping of LTSS in the RMC utilizing the 157 markers (148 SSRs) genotypes that were available at that time. Subsequently, the RMC was resequenced, which significantly improved the marker density (Wang et al., 2016; Huggins et al., 2019). For this study, the LTSS phenotypes from five trials (Schläppi et al., 2017) were rerun utilizing the denser SNP genotypes. Eight panels were used to capture the genetic diversity present in specific subpopulations and groups. All panels were filtered to exclude heterozygous markers and markers with a minor allele frequency of less than 5%. The panel “All Lines” contained all 173 RMC accessions genotyped with 3,224,845 SNPs. For the subpopulation panels, IND had 58 accessions genotyped with 1,883,603 SNPs, AUS had 30 accessions genotyped with 1,753,773 SNPs, TRJ had 28 accessions genotyped with 1,067,722 SNPs, and TEJ had 30 accessions genotyped with 691,001 SNPs. The ARO subpopulation only contained 11 accessions, so it was not an individual panel but was included in the “All Lines” panel and in the JAPARO panel described below. Panels of combined subpopulations were *Indica* (INDAUS) composed of IND and AUS having 92 accessions genotyped with 2,477,397 SNPs, *Japonica* (JAP) composed of TRJ and TEJ having 61 accessions genotyped with 1,092,889 SNPs, and JAPARO composed of JAP and ARO having 77 accessions genotyped with 1,402,000 SNPs. A mixed linear model was used in Tassel V 5.0 (Bradbury et al., 2007) for GWAS. GWAS run conditions, PCA, and kinship matrix are summarized in Eizenga et al. (2023).

GWAS results were run through Perl scripts to identify associated chromosome regions from individual SNPs or groups of physically linked SNPs (Huggins et al., 2019). Chromosome regions were set to 50 kilobases (kb) in both directions around each individual significant SNP and were extended to include nearby significant SNPs occurring within 200 kb. A Perl script was used to designate a “Peak SNP” for each region, which corresponded to the SNP with the most significant *p*-value found within the region.

### *In silico* analysis of candidate genes within mapped QTL

Genomic variations of single nucleotide polymorphisms (SNPs) and insertion/deletions (INDELs) between parents of the RIL and BIL populations were determined manually using the RiceVarMap database (http://ricevarmap.ncpgr.cn; Zhao et al., 2015). Information about cold temperature-regulated gene expression was retrieved from the RNA-seq PPRD database containing 11,726 rice libraries (http://ipf.sustc.edu.cn/pub/plantrna/; Yu et al., 2022).

## Results and discussion

### Description of RIL and BIL populations

**Figure 1:**
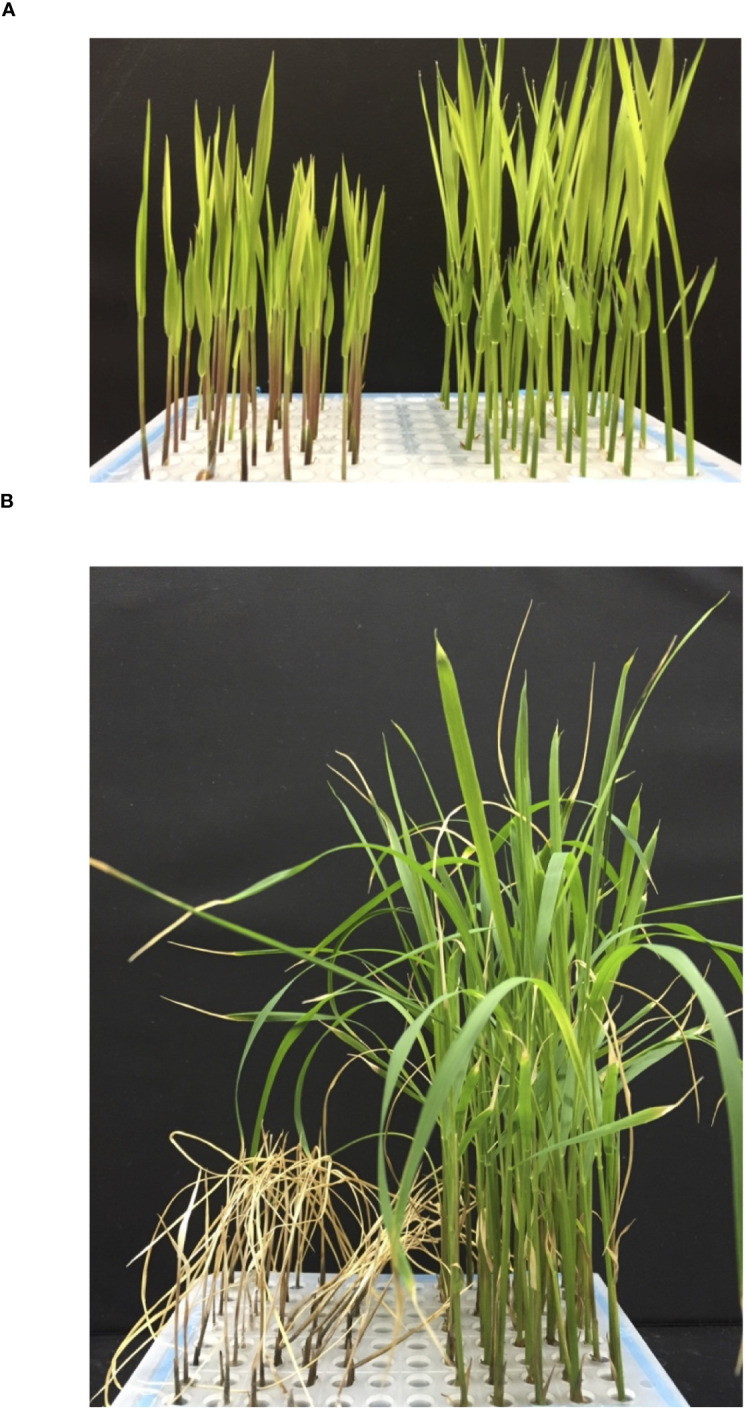
Phenotypes of two rice varieties with varying cold tolerance potentials. **(A)** Two-week-old hydroponically grown rice seedlings. The cold-sensitive control Carolino 164 (*aus*) is shown on the left (red seedlings), and the cold-tolerant check Krasnodarskij 3352 (*temperate japonica*; green seedlings) is shown on the right. **(B)** Phenotype after 1 week of 10°C exposure and 1 week of warm temperature recovery growth.

Four overall cold-tolerant (CT) TEJ accessions and four overall cold-sensitive (CS) accessions from the *Indica* VG, including AUS and IND accessions, were selected from the RMC-based GWAS mapping of the RMC for cold tolerance (Schläppi et al., 2017). Crosses were attempted between four pairs of these CT and CS accessions. **Figure 1** shows the phenotypes of a pair of CS and CT parents where 2-week-old seedlings were exposed for 1 week to constant chilling temperatures of 10°C, after which they were allowed to recover at warm temperatures for 1 week. In the example shown, most CS seedlings did not survive; they were bleached and started to droop during the recovery period. Conversely, most of the CT seedlings survived, remained green, and resumed growth and development during the recovery period.

Due to major flowering time and sterility issues with two of the pairs, only two crosses were selected for further population development. A recombinant inbred line (RIL) mapping population was developed from CT Krasnodarskij 3352 (TEJ) crossed with CS Carolino 164 (AUS), advanced by single-seed descent to the F_8:9_ generation, for a final population of 140 RILs. To generate a backcross inbred line (BIL) mapping population with reduced sterility issues, WIR 911 (TEJ) was crossed with Carolino 164, and F_1_ plants were backcrossed as female with Carolino 164. The backcross progeny was advanced by single-seed descent to the BC_1_F_4:5_, for a final population of 93 BILs. Leaf tissue from single plants of the 140 F_7:8_ RILs and 93 BC_1_F_4:5_ BILs was genotyped, and after curation, more than 2,500 SNPs were retained. For QTL mapping of the RIL population, 2,794 SNPs with an average of 233 SNPs per chromosome (range: 170–346 SNPs) were used (**Supplementary Table S3**), and for BIL population mapping, 2,590 SNPs with an average of 216 SNPs per chromosome (range: 154–324 SNPs) were used (**Supplementary Table S4**). For both populations, chromosome (chr.) 9 had the fewest, and chr. 1 the most SNPs.

### Mapping potential validation of RIL and BIL populations

**Figure 2:**
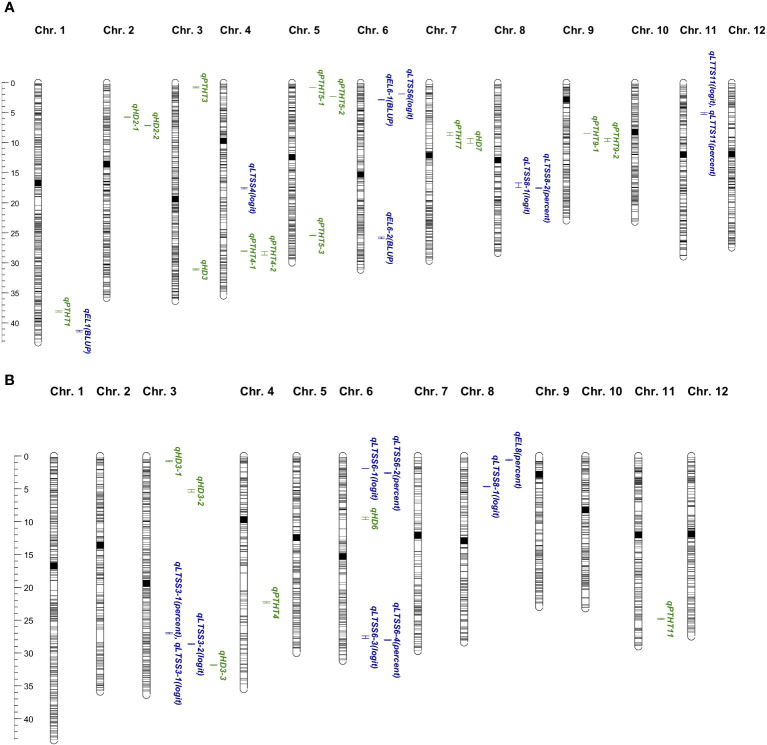
**(A)** Recombinant inbred line (RIL) quantitative trait loci (QTL) map in Mb. Shown are all 12 rice chromosomes with 2,794 single nucleotide polymorphism (SNP) markers as gray horizontal lines (centromere regions in black). In total, 134 Krasnodarskij 3352 × Carolino 164 RIL F_8:9_ progeny were used for mapping the agronomic traits (shown in green), heading date (*qHD*), and plant height (*qPTHT*) and for mapping the cold tolerance traits (shown in blue), percent electrolyte leakage (*qEL*), and percent low-temperature survivability (*qLTSS*). [The map was created with MapChart 2.3 (Voorrips, 2002)]. **(B)** Backcross recombinant inbred line (BIL) quantitative trait loci (QTL) map in Mb. Shown are all 12 rice chromosomes with 2,590 single nucleotide polymorphism (SNP) markers as gray horizontal lines (centromere regions in black). A total of 92 (WIR 911 x Carolino 164) x Carolino 164 BIL F_4:5_ progeny were used for mapping the agronomic traits (shown in green), heading date (*qHD*), and plant height (*qPTHT*) and for mapping the cold tolerance traits (shown in blue), percent electrolyte leakage (*qEL*), and percent low-temperature survivability (*qLTSS*). [The map was created with MapChart 2.3 (Voorrips, 2002)].

To perform a “quality control” for the bi-parental mapping potential of the RIL and BIL populations, two-week-old chamber-grown seedlings were transplanted into raised bed rooftop paddies at Marquette University (43°02′11.59″ N/87°55′51.64″ W). Heading date (HD) and plant height (PTHT) data indicated that the parents were at the extremes of each end of the normal HD distributions, with only a few “transgressive” plants, while the normal PTHT distributions showed increased transgressive variation (**Supplementary Figures S1A, B**; **Tables 1**, **2**). QTL mapping of HD and PTHT QTL revealed eight *qHD* and 12 *qPTHT* with logarithm of odds (LOD) scores >3.0 (**Table 3**). The general locations of the RIL HD QTL, *qHD3* and *qHD7*, and BIL HD QTL, *qHD3-1*, *qHD3-2*, *qHD3-3*, and *qHD6* (**Figure 2**) matched previously obtained legacy HD QTL (Takahashi et al., 2001), which validated the RIL and BIL populations for QTL mapping.

**Table 1:** Summary statistics (overall mean, SE, range of progeny) of the four traits measured on the 134 Krasnodarskij 3352 × Carolino 164 progeny lines included in the quantitative trait locus analysis (**Tables 3**, **4**) and parents.

Trait (acronym)	Recombinant inbred line progeny			Parents	
	Mean	SE^a^	Range	Krasnodarskij 3352	Carolino 164
Agronomic traits					
Heading date (HD) (days)^b^	90.4	0.5	76–99	77.5	96.7
Plant height (PTHT) (cm)	79.4	1.6	51.0–121.3	101.6	95.7
Chilling tolerance traits					
Electrolyte leakage (EL) (%)	22.8	1.3	9.8–75.2	14.6	39.7
Low-temperature seedling survivability (LTSS) (%)	82.1	1.5	9.1–100.0	100.0	8.7

Trait distributions are shown in **Supplementary Figure S1A**.

a The SE of the mean was calculated as the SD divided by the square root of the number of entries.

b Number of days from germination to panicle emergence.

**Table 2:** Summary statistics (overall mean, SE, range of progeny) of the four traits measured on the 92 (WIR 911 × Carolino 164) × Carolino 164 backcrossed progeny lines included in the quantitative trait locus analysis (**Tables 3**, **4**) and parents.

Trait (acronym)	Backcrossed inbred line progeny			Parents	
	Mean	SE^a^	Range	WIR 911	Carolino 164
Agronomic traits					
Heading date (HD) (days)^b^	89.2	0.9	70–120	67.2	96.7
Plant height (PTHT) (cm)	91.0	1.4	59.0–117.7	88.9	95.7
Chilling tolerance traits					
Electrolyte leakage (EL) (%)	28.8	1.2	13.1–72.3	17.5	34.1
Low-temperature seedling survivability (LTSS) (%)	50.5	3.4	0–100	99.2	11.6

Trait distributions are shown in **Supplementary Figure S1B**.

a The SE of the mean was calculated as the SD divided by the square root of the number of entries.

b Number of days from germination to panicle emergence.

**Table 3:** List of the heading date (HD) and plant height (PTHT) quantitative trait loci (QTL) identified in the Krasnodarskij 3352 × Carolino 164 recombinant inbred line (RIL) and (WIR 911 × Carolino164) × Carolino164 backcross inbred line (BIL) mapping populations.

QTL^a^	Chr.	QTL region (cM)	QTL interval (Mb)	Marker nearest LOD peak	Peak position (cM)	LOD value	Additive effect^b^	PVE^c^
Krasnodarskij 3352 × Carolino 164 RIL population								
*qHD2-1*	2	34.3–35.3	5.72–5.84	2_5837031	35.0	5.7	−1.64	7.5
*qHD2-2*	2	41.3–41.8	7.15–7.24	2_7238793	41.5	10.6	2.31	14.8
*qHD3*	3	142.3–142.8	31.02–31.19	3_31190989	142.5	5.3	−1.54	6.5
*qHD7*	7	47.3–48.3	9.26–10.11	7_10111835	48.0	16.6	−3.27	28.3
*qPTHT1*	1	149.8–150.8	38.05–38.28	1_38239037	150.0	10.4	−4.82	5.7
*qPTHT3*	3	2.36–3.14	0.73–0.85	3_851612	3.0	6.9	−3.84	3.4
*qPTHT4-1*	4	80.3–82.3	28.01–28.10	4_28098691	80.5	12.3	−5.88	7.9
*qPTHT4-2*	4	82.3–83.8	28.01–28.69	4_28685882	83.5	7.8	4.68	4.8
*qPTHT5-1*	5	4.26–5.43	0.77–0.81	5_805425	5.0	6.5	−3.67	3.3
*qPTHT5-2*	5	16.3–17.8	2.27–2.29	5_2291156	16.5	15.2	6.29	9.6
*qPTHT5-3*	5	87.3–88.3	25.44–25.54	5_25502053	87.5	12.1	−5.32	7.0
*qPTHT7*	7	43.8–46.3	8.32–8.81	7_8806939	44.5	19.6	−7.69	14.1
*qPTHT9-1*	9	11.3–11.8	8.51–8.52	9_8515797	11.5	23.1	−8.98	18.6
*qPTHT9-2*	9	16.3–17.3	9.33–9.78	9_9778764	17.5	10.0	4.91	5.4
(WIR 911 × Carolino164) × Carolino164 BIL population								
*qHD3-1*	3	0.0–0.3	0.73–0.85	3_730996	0.0	14.9	−4.56	27.8
*qHD3-2*	3	16.3–19.3	5.09–5.54	3_5534861	17.0	3.3	−1.82	4.5
*qHD3-3*	3	100.3–101.3	31.76–31.89	3_31893660	101.0	3.2	−1.95	4.5
*qHD6*	6	18.3–20.3	9.32–9.69	6_9314865	19.0	15.3	6.17	28.8
*qPTHT4*	4	45.3–46.3	22.23–22.44	4_22231867	46.0	3.5	−4.40	12.2
*qPTHT11*	11	64.3–65.3	24.73–24.93	11_24728131	65.5	3.0^d^	−6.79	10.8

The RIL population had 134 progeny genotyped with 2,794 SNP markers and the BIL population had 92 progeny genotyped with 2,590 SNP markers.

a Quantitative trait loci (QTL) are declared based on the Inclusive Composite Interval Mapping (ICIM) approach using the IciMapping software (Meng et al., 2015) and 0.5-step, 0.005-pin ICIM_Add settings unless otherwise noted.

b A positive additive effect indicates that the Krasnodarskij 3352 or WIR 911 allele increases the phenotype for that trait; a negative additive effect indicates that the Carolino164 allele increases the trait.

c PVE is the percentage of total phenotypic variation explained by an individual QTL, as estimated by R^2^ values from ICIM analysis.

d LOD score is close to the 90% confidence value of 3.01 (based on 1,000 permutations for the PTHT trait).

The fact that six *qHD* overlapped with or were near known rice heading date genes provided additional validation. *qHD3-1*, *qHD6*, and *qHD7* had LOD scores of 14.9, 15.3, and 16.6, explained 27.8%, 28.8%, and 28.3% of the observed variation, and uncovered the major heading date genes, *Ehd4*, *Hd1*, and *Ghd7*, respectively (**Table 3**; **Supplementary Table S5**; Yano et al., 2000; Xue et al., 2008; Gao et al., 2013). Those three genes have previously identified high-impact SNP variants that might explain the major heading date differences of the three RIL and BIL parents (**Supplementary Table S6**; **Tables 1**, **2**; Xue et al., 2008; Ishikawa et al., 2011; Gao et al., 2013; Wei et al., 2021). *qHD3*, *qHD3-2*, and *qHD3-3* with minor heading date effects overlapped with previously identified heading date genes *OsMADS14* (Wei et al., 2020), *OsIDD1* (Colasanti et al., 2006), and *OsTPR075* (Zhang et al., 2022), and *Hd6* (Takahashi et al., 2001), respectively, and promoter or coding region SNP variants might contribute to heading date differences between the parents (**Supplementary Tables S5**, **S6**). Taken together, HD QTL mapping uncovered previously identified heading date genes, thus validating the RIL and BIL populations for QTL mapping.

This conclusion was further supported by plant height QTL mapping. Of the 10 PTHT QTL, two contained known genes affecting plant height (**Supplementary Table S5**). *qPTHT1* and *qPTHT5-2* uncovered the chitin-inducible gibberellin-responsive gene, *plant height 1* (*ph1*), and the gibberellin 2-beta-dioxygenase gene, *OsGAox4*, respectively, and nonsynonymous amino acid substitutions might contribute to plant height differences between the parents (**Supplementary Table S6**; Kovi et al., 2011; He et al., 2022). Taken together, heading date and plant height QTL mapping using the newly developed RIL and BIL populations described here yielded several QTL with high LOD scores containing known flowering time and plant height-regulating genes, thus validating their usefulness for mapping cold tolerance QTL and narrowing down associated cold tolerance genes.

### Cold tolerance trait mapping

EL assays were done on 2-week-old seedlings after 1 week of exposure to 10°C as a measure of plasma membrane integrity right after the cold temperature exposure. After 1 week of recovery growth, LTSS was determined as a measure of the ability of cold-stressed plants to maintain photosynthesis and resume growth and development. The chilling temperature and exposure time were chosen, because based on previous studies (Schläppi et al., 2017), the cold-tolerant parents had >90% survival while the cold-sensitive parent had approximately 10% survival, thus avoiding a scenario where most cold-sensitive RILs and BILs would have 0% survival. The RIL mapping yielded nine QTL with LOD scores of 2.7 or higher (three EL and five unique LTSS QTL; **Table 4**), and the BIL mapping also yielded nine QTL with LOD scores of 3.2 or higher (one EL and seven unique LTSS QTL; **Table 4**). Of the 16 unique cold tolerance trait QTL, 11 were found in clusters on chr. 3, chr. 6, and chr. 8, while a single QTL was found on chr. 1, chr. 4, and chr. 11 (**Figure 2**).

**Table 4:** List of cold tolerance quantitative trait loci (QTLs) for the traits electrolyte leakage (EL) and low-temperature survivability (LTSS) identified in the Krasnodarskij 3352 × Carolino 164 recombinant inbred line (RIL) and (WIR 911 × Carolino164) × Carolino164 backcross inbred line (BIL) mapping populations.

QTL^a^	Chr.	QTL region (cM)	QTL interval (Mb)	Marker nearest LOD peak	Peak position (cM)	LOD value	Additive effect^b^	PVE^c^
Krasnodarskij 3352 × Carolino 164 RIL population								
*qEL1*(BLUP)	1	175.8–177.8	41.19–41.54	1_41191016	176.5	3.0	−2.92	8.9
*qEL6-1*(BLUP)	6	10.8–12.3	2.78–2.87	6_2783620	11.0	3.1	−2.48	9.1
*qEL6-2*(BLUP)	6	92.9–96.3	25.73–25.96	6_25732013	93.0	2.7^d^	−2.28	8.0
*qLTSS4*(logit)	4	17.3–24.3	17.47–17.66	4_17467268	23.0	2.8^e^	0.34	6.7
*qLTSS6*(logit)	6	6.3–6.8	1.87–1.93	6_1867779	6.5	3.2	0.37	7.9
*qLTSS8-1*(logit)	8	57.3–59.8	16.69–17.52	8_17516403	59.5	4.4	0.44	11.2
*qLTSS8-2*(percent)	8	59.8–60.3	17.52–17.58	8_17575726	60.0	2.7^f^	4.23	8.5
*qLTSS11*(percent)	11	59.8–60.3	5.03–5.34	11_5339007	25.5	3.4	4.77	10.8
*qLTSS11*(logit)	11	23.3–26.3	5.03–5.34	11_5339007	26.0	3.7	0.39	9.2
(WIR 911 × Carolino164) × Carolino164 BIL population								
*qEL8*(percent)	8	0.0–0.3	0.53–0.66	8_658032	0.0	4.3	−4.60	15.4
*qLTSS3-1*(percent)	3	77.8–79.8	26.81–27.07	3_26919429	79.0	3.2^g^	−8.47	11.5
*qLTSS3-1*(logit)	3	78.3–79.8	26.81–27.07	3_27066394	79.5	11.3	−0.98	21.1
*qLTSS3-2*(logit)	3	84.8–86.8	28.55–28.99	3_28688032	85.5	4.5	0.57	7.0
*qLTSS6-1*(logit)	6	1.8–2.3	1.87–1.93	6_1867779	2.0	7.0	0.97	11.7
*qLTSS6-2*(percent)	6	2.3–2.8	2.55–2.75	6_2747409	2.5	3.9	12.89	13.7
*qLTSS6-3*(logit)	6	49.8–51.1	27.37–27.76	6_27761109	51.0	5.5	0.77	9.1
*qLTSS6-4*(percent)	6	53.3–53.8	28.02–28.06	6_28023780	53.5	4.2	11.76	15.3
*qLTSS8-1*(logit)	8	15.3–15.8	4.60–4.70	8_4699206	15.5	3.6	0.61	5.6

The RIL population had 134 progeny genotyped with 2,794 SNP markers and the BIL population had 92 progeny genotyped with 2,590 SNP markers.

a Quantitative trait loci (QTL) are declared based on the Inclusive Composite Interval Mapping (ICIM) approach using the IciMapping software (Meng et al., 2015) and 0.5-step, 0.005-pin ICIM_Add settings unless otherwise noted.

b A positive additive effect indicates that the Krasnodarskij 3352 or WIR 911 allele increases the phenotype for that trait; a negative additive effect indicates that the Carolino164 allele increases the trait.

c PVE is the percentage of total phenotypic variation explained by an individual QTL, as estimated by R^2^ values from ICIM analysis.

d LOD score is close to the 90% confidence value of 2.774 (based on 1,000 permutations for the EL(BLUP) trait).

e LOD score is >90% confidence value of 2.858 and <95% value of 3.205 (based on 1,000 permutations for the LTSS(logit) trait).

f LOD score is close to the 90% confidence value of 2.876 (based on 1,000 permutations for the LTSS(percent) trait).

g LOD score is close to the 95% confidence value of 3.384 (based on 1,000 permutations for the LTSS(percent) trait).

Strikingly, all 16, in other words, 100% of the QTL (**Supplementary Table S7**) overlapped with seedling stage cold tolerance GWAS QTL discovered in the RMC or RDP1 (Schläppi et al., 2017; Shimoyama et al., 2020; Phan and Schläppi, 2021). Of note, for this study, GWAS QTL for the RMC was mapped more precisely using the phenotypes reported by Schläppi et al. (2017) and genotypes based on 3,224,845 SNPs (Wang et al., 2016; Huggins et al., 2019). Similarly, the precision of the GWAS mapping in the RDP1 was improved using the seedling stage cold tolerance phenotypes reported by Shimoyama et al. (2020) and genotypes based on 4,829,392 SNPs (Wang et al., 2018). These 16 overlapping QTL were analyzed to identify cold tolerance genes within the QTL regions.

The EL and LTSS QTL identified using the biparental RIL and BIL populations were smaller than those found by GWAS mapping in the RMC and RDP1, thus reducing the number of genes to investigate (**Table 5**; Schläppi et al., 2017; Shimoyama et al., 2020; Phan and Schläppi, 2021). Therefore, an analysis of genomic variation of nontransposable element genes between the CT and CS parents using a public database (Zhao et al., 2015) and literature searches were done to narrow down cold tolerance candidate genes within the QTL regions and 100,000 bp to the left and right marker SNPs, which uncovered 25 cold tolerance candidate genes in 15 QTL (**Table 5**). The SNP ID numbers, nucleotide changes, and SNP impacts in alleles of the cold-sensitive parent Carolino 164, such as nonsynonymous amino acid changes, frameshifts, premature stop, loss of start, loss of stop, and splice variants, are summarized in **Table 6**.

**Table 5:** The quantitative trait loci (QTL) identified in the Rice Mini-core collection and Rice Diversity Panel 1 genome-wide association studies (GWAS) reported in **Supplementary Table S7** that colocalized with Krasnodarskij 3352 × Carolino 164 RIL or (WIR 911 × Carolino164) × Carolino164 BIL QTL regions (**Table 3**) for chilling tolerance.

QTL	Chr.	RIL/BIL QTL interval (Mb)^a^	GWAS QTL region (Mb)	Gene symbol^b^	Position (Mb)^c^	Loc ID^d^	Citation
Krasnodarskij 3352 × Carolino 164 RIL population							
Electrolyte leakage							
*qEL1*(BLUP)	1	41.19–41.54	41.16–41.26	*OsACA6*	41.22	LOC_Os01g71240	Huda et al. (2014)
*qEL6-1*(BLUP)	6	2.78–2.87	0.80–2.80	*OsFBL27*	2.78	LOC_Os06g06050	Tang and Thompson (2019)
			2.88–2.98	(esterase)	2.80	LOC_Os06g06080	This study
				*OsSIPK/OsMPK6*	2.81	LOC_Os06g06090	Lee et al. (2008)
*qEL6-2*(BLUP)	6	25.73–25.96	25.71–26.67				
Low-temperature seedling survivability							
*qLTSS4*(logit)	4	17.47–17.66	17.05–17.57	*OsWAK38*	17.67	LOC_Os04g29680	Wang et al. (2021)
			17.51–17.61	*OsWAK40*	17.75	LOC_Os04g29790	Wang et al. (2021)
*qLTSS6*(logit)	6	1.87–1.92	0.80–2.80	*OsRLK-803* (transferase)	1.87	LOC_Os06g04370	This study
					1.88	LOC_Os06g04380	Norton et al. (2008)
*qLTSS8-1*(logit)	8	16.69–17.52	16.37–17.31	*LTPL130*	16.86	LOC_Os08g27674	Padmashree et al. (2023)
*qLTSS8-2*(percent)	8	17.52–17.58	17.46–18.18	*OsRLCK253*	17.56	LOC_Os08g28710	Le et al. (2021)
*qLTSS11*(percent)	11	5.03–5.34	4.92–5.37	*OsFBX398*	5.03	LOC_Os11g09360	Higgins et al. (2022)
*qLTSS11*(logit)	11	5.03–5.34	4.71–6.71	*OsPDIL1-1*	4.97	LOC_Os11g09280	Xia et al. (2018)
(WIR 911 × Carolino164) × Carolino164 BIL population							
Electrolyte leakage							
*qEL8*(BLUP)	8	0.53–0.66	0.64–1.41	*RF6*	0.54	LOC_Os08g01870	Huang et al. (2015)
Low-temperature seedling survivability							
*qLTSS3-1*(percent)	3	26.81–27.07	26.65–27.21	*OsANK*	26.82	LOC_Os03g47460	Huang et al. (2009)
*qLTSS3-1*(logit)	3	26.81–27.07	26.96–27.81	*OsANK*	27.01	LOC_Os03g47686	Huang et al. (2009)
			27.02–27.20	*OsANK*	27.02	LOC_Os03g47693	Huang et al. (2009)
				*OsANK*	27.04	LOC_Os03g47720	Huang et al. (2009)
*qLTSS3-2*(logit)	3	28.55–28.99	28.41–28.65	*OsGST3*	28.60	LOC_Os03g50130	Chen et al. (2023)
*qLTSS6-1*(logit)	6	1.87–1.93	0.80 - 2.80	*OsRLK-803*	1.87	LOC_Os06g04370	This study
				(transferase)	1.88	LOC_Os06g04380	Norton et al. (2008)
*qLTSS6-2*(percent)	6	2.55–2.75	0.80–2.80	*OsNAPL2*	2.56	LOC_Os06g05660	(Tripathi et al. (2015); Tripathi et al. (2016)
			2.41–2.71	(transferase)	2.63	LOC_Os06g05790	This study
*qLTSS6-3*(logit)	6	27.37–27.90	26.85–27.80	*OsFKBP13*	27.40	LOC_Os06g45340	Ahn et al. (2010)
			27.60–29.60	*CYP59A*	27.78	LOC_Os06g45900	Ahn et al. (2010)
*qLTSS6-4*(percent)	6	28.02–28.06	27.80–28.72	*OsOMTN4/NAC011*	28.04	LOC_Os06g46270	Fang et al. (2014)
			27.60–29.60	(hydrolase)	28.05	LOC_Os06g46284	This study
*qLTSS8*(logit)	8	4.60–4.70	4.36–5.06	*OsbHLH070*	4.65	LOC_Os08g08160	Li et al. (2006)

The candidate genes located in the QTL regions are listed.

a The Mb position of the flanking markers for the RIL or BIL QTL interval is based on the Mb position of the markers that were closest to the cM position of the QTL region as defined by the QTL analysis and listed in **Table 3**.

b Gene nomenclature followed the standardized nomenclature for rice genes used in Oryzabase (Yamazaki et al., 2010). The Committee on Gene Symbolization, Nomenclature, and Linkage gene symbol was used, if available.

c The pseudomolecule Mb position of the candidate gene in the Rice Genome Annotation Project Release 7 (Kawahara et al., 2013).

d Rice Genome Annotation Project locus identifier for the candidate gene (Kawahara et al., 2013).

**Table 6:** High impact SNP variants in cold-sensitive *aus* line Carolino 164 compared to cold-tolerant *japonica* line Krasnodarskij 3352.

Chr.	Gene annotation	Locus ID	SNP ID	Nucleotide change	Impact
1	*OsACA6*	LOC_Os01g71240	na	na	na
3	*OsANK*	LOC_Os03g47640	vg03_26984941	C>T	Arg355^*^
3	*OsANK*	LOC_Os03g47686	vg03_27020751	C>G	Tyr518^*^
3	*OsANK*	LOC_Os03g47693	vg03_27022097	G>A	Trp56^*^
3	*OsANK*	LOC_Os03g47720	vg03_27044642	CAA>CAAA	Glu86fs
3	*OsGST3*	LOC_Os03g50130	vg03_28595867	A>G	start_lost;Met1?
7	*OsWAK38*	LOC_Os04g29680	na	na	na
4	*OsWAK40*	LOC_Os04g29790	vg04_17752550	A>AAC	Ile158fs^a^
			vg04_17752554	TTG>T	Lys157fs^a^
6	*OsRLK-803*	LOC_Os06g04370	vg06-01867369	A>T	Ser529Cys
6	Transferase	LOC_Os06g04380	vg06_01875711	A>AC	Ser56fs
6	*OsNAPL2*	LOC_Os06g05660	vg06_02560533	G>T	Gly126Cys
6	Transferase	LOC_Os06g05790	vg06_02633463	T>TA	Leu483fs
6	*OsFBL27*	LOC_Os06g06050	vg06_02780806	A>AGAG	Glu6dup
			vg06_02781107	TGCGGCGGCGGCG>TGCGGCGGCGGCGGCG	Glu110dup
6	Esterase	LOC_Os06g06080	vg06_02803110	TCC>T	Arg25fs
6	*OsMPK6*	LOC_Os06g06090	vg06_02812762	GCCC>G	Gly29del^b^
6	*OsFKBP13*	LOC_Os06g45340	vg06_27403638	CGG>N/C	Ala4fs (if C)
6	*CYP59A*	LOC_Os06g45900	vg06_27783122	C>CGGGG	Leu43fs
			vg06_27791008	T>C	Ter549Arg^ext*?^
6	*OsNAC011*	LOC_Os06g46270	vg06_28037986	GCGGGCGGCG>C	Ala38_Ala50del
6	Hydrolase	LOC_Os06g46284	vg06_28057827	A>T	Tyr74^*^
8	*RF6*	LOC_Os08g01870	vg08_00538004	T>C	Cys484Arg
8	*OsbHLH070*	LOC_Os08g08160	vg08_04647095	T>TAA	Ile333fs
			vg08_04648125	CT>C	Splice variant
8	*LTPL130*	LOC_Os08g27674	vg08_16869517	TG>T	Thr61fs
8	*OsRLCK253*	LOC_Os08g28710	vg08_17559305	T>TCGC	Ala256 dup
11	*OsPDIL1-1*	LOC_Os11g09280	vg11_04974896	C>CG	Gly35fs
			vg11_04974890	GCATC>G	Asp36fs
11	*OsFBX398*	LOC_Os11g09360	vg11_05026584	G>A	Splice variant

na, not available; ext^*^?, stop codon lost, reading frame extended; fs, frameshift.

^*^Premature stop codon.

a Variant in Krasnodarskij 3352; Carolino 164 same as the Nipponbare reference genome.

b Total of 233 variants between Krasnodarskij 3352 and Carolino 164.

Three of the four EL QTLs on chr. 1 (*qEL1*), chr. 6 (*qEL6-1*), and chr. 8 (*qEL8*) had five cold tolerance candidate genes associated with them: the P-type IIB Ca^2+^ ATPase gene *OsACA6* (LOC_Os01g71240), the F-box protein gene *OsFBL27* (LOC_Os06g06050), the serine esterase encoding gene LOC_Os06g06080, the mitogen-activated protein kinase gene *OsMPK6* (LOC_Os06g06090), and the pentatricopeptide repeat type restorer-of-fertility gene *RF6* (LOC_Os08g01870). *OsACA6* was previously shown to enhance cold tolerance in transgenic tobacco (Huda et al., 2014), and due to nucleotide variations in the promoter region, there might be differences in gene expression levels between CT and CS accessions. *OsFBL27* activates *OsMYB30*, a negative regulator of cold tolerance, and *OsFBL27* is downregulated by *OsmiR528* (Tang and Thompson, 2019). Observed amino acid duplications and added nucleotides in the CS parent might enhance *OsMYB30* activation due to increased *OsFBL27* activity and/or lack of *OsmiR528*-mediated downregulation. *OsMPK6* was previously shown to be cold inducible (Lee et al., 2008), and the 233 SNP variants between Carolino 164 and Krasnodarskij 3352 might affect kinase activity or regulation. Previously, other Ca^2+^ ATPase, F-box protein, and mitogen-activated protein kinase genes were shown to be involved in cold stress tolerance response mechanisms leading to the production of compatible osmolytes that might stabilize biological membranes (reviewed in Li et al., 2022). The candidate genes uncovered here might have similar functions, which need to be tested in future studies. *RF6* might be involved in nucleotide and nucleic acid metabolic processes in the mitochondrion (Huang et al., 2015), affecting reactive oxygen species (ROS) production, while the involvement of serine esterases in cold tolerance needs to be addressed in future studies.

The remaining 20 candidate genes were associated with 11 LTSS QTL, as follows: The two QTL on chr. 3 (*qLTSS3-1* and *qLTSS3-2*) had four ankyrin-tetratricopeptide repeat (ANK-TPR) genes (*OsANK*; LOC_Os03g47460, LOC_Os03g47686, LOC_Os03g47693, LOC_Os03g47720) and the microsomal glutathione *S*-transferase gene *OsGST3* (LOC_Os03g50130). The single QTL on chr. 4 (*qLTSS4*) had the two wall-associated kinase genes *OsWAK38* (LOC_Os04g29680) and *OsWAK40* (LOC_Os04g29790). The four QTL on chr. 6 (*qLTSS6-1*, *qLTSS6-2*, *qLTSS6-3*, *qLTSS6-4*) had eight candidate genes: the receptor-like kinase gene *OsRLK-803* (LOC_Os06g04370); the aminomethyltransferase gene LOC_Os06g04380; the histone chaperone gene *OsNAPL2* (LOC_Os06g05660); the transferase domain-containing protein gene LOC_Os06g05790; the two peptidyl-prolyl *cis*/*trans* isomerase genes *OsFKBP13* (LOC_Os06g45340) and *CYP59A* (LOC_Os06g45900); and the NAC transcription factor gene *OsNAC011* (LOC_Os06g46270) and the glycosyl hydrolase family 31 gene LOC_Os06g46284. The three QTL on chr. 8 (*qLTSS8*, *qLTSS8-1*, *qLTSS8-2*) had the transcription factor gene *OsbHLH070* (LOC_Os08g08160), the protease inhibitor/seed storage/LTP family protein precursor gene *LTPL130* (LOC_Os08g27674), and the receptor-like cytoplasmic kinase gene *OsRLCK253/CRINKLY4* (LOC_Os08g28710). The single QTL on chr. 11 (*qLTSS11*) had the F-box genes *OsFBX398* (LOC_Os11g09360) and the protein disulfide isomerase gene *OsPDIL1-1* (LOC_Os11g09280). These 20 genes can be categorized into three general groups: (1) genes with kinase activity potentially involved in cold-mediated signal transduction; (2) genes that potentially regulate gene expression; and (3) genes that code for potential cold tolerance effectors such as chaperones or enzymes.

Many of the 20 cold tolerance candidate genes were previously shown to be involved in stress tolerance responses. Specifically, in the kinase category, *OsWAK38* and *OsWAK40* upregulation was specifically shown to be associated with salt tolerance (Wang et al., 2021), and *OsRLCK253* was previously shown to be the only candidate gene associated with QTL_19 (Le et al., 2021) because of its involvement in water-deficit improvement and salinity stress tolerance (Giri et al., 2011). In the gene expression category, *OsNAC011* was specifically shown to regulate drought tolerance in rice (Fang et al., 2014), and *OsFBX398* was previously identified as a candidate gene selected during the breeding of rice for adaptation to different environments in Vietnam (Higgins et al., 2022). In the potential cold tolerance effector category, chaperone-type *ANK* genes were shown to have numerous functions in plants (Huang et al., 2009), including roles in response to abiotic and biotic stresses, and histone chaperone *NAP* genes were previously shown to have a role in abiotic stress responses (Tripathi et al., 2016). For enzyme-encoding genes, *OsGST3* was specifically shown to respond to various stress hormones (Chen et al., 2023), and *OsPDIL1-1* overexpression in transgenic rice was shown to enhance abiotic stress tolerance (Xia et al., 2018), possibly by controlling the production of ROS (Zhao et al., 2023). It was also shown that the aminomethyltransferase gene LOC_Os06g04380 was differentially expressed after arsenic treatment (Norton et al., 2008), that peptidyl-prolyl *cis*/*trans* isomerase genes have various functions in response to environmental stresses (Ahn et al., 2010), and that *LTPL130* was previously selected as a candidate gene located on chr. 8 at 16.86 Mb in a QTL, a QTL for several root and yield-associated traits under aerobic and irrigated conditions (Padmashree et al., 2023).

Besides allelic differences between CT and CS rice accessions, another criterion for cold tolerance candidate genes is that their steady-state mRNA levels are up- or downregulated in response to cold temperatures. Strikingly, a search of publicly available databases determined that of the 24 candidate genes with expression data, 22, in other words, 91.7% were regulated by cold temperatures (**Table 7**). Of those 22, eight (i.e., 36.4%) were up- and 11 (i.e., 50.0%) were downregulated, while three (i.e., 13.6%) were either up- or downregulated in different data sources. Because genetic backgrounds, degree of cold, and lengths of cold exposures were different between data sets (**Supplementary Table S8**), it will be necessary to reexamine expression patterns of the cold tolerance candidate genes under the conditions we used for QTL mapping. However, it is conceivable that stably up- and downregulated genes might have positive and negative effects, respectively, in cold stress tolerance responses in rice. Such genes might be differentially regulated in CT and CS rice accessions, which, together with coding region SNP variants, would be another reflection of allelic differences between accessions with varying cold tolerance potentials.

**Table 7:** Cold temperature-regulated gene expression of QTL-associated candidate genes.

Chr.	Position (Mb)	Gene annotation	Locus ID	Fold change	Data source^a^
1	41.22	*OsACA6*	LOC_Os01g71240	2.9; 3.9; 4.1; 5.8	PRJNA610422
				−3.8	PRJNA281699
3	26.82	*OsANK*	LOC_Os03g47640	4.2	PRJNA610422
3	27.01	*OsANK*	LOC_Os03g47686	−3.5; −1.5; −0.78	PRJDB6837
3	27.02	*OsANK*	LOC_Os03g47693	1.5	PRJNA564250
3	27.04	*OsANK*	LOC_Os03g47720	ns	
3	28.60	*OsGST3*	LOC_Os03g50130	−1.3; −2.7	PRJDB2600
4	17.67	*OsWAK38*	LOC_Os04g29680	8.1; 2.8	PRJNA448164
4	17.75	*OsWAK40*	LOC_Os04g29790	−23.2	PRJDB2600
6	1.87	*OsRLK-803*	LOC_Os06g04370	−2.5; −4.7	PRJNA610422
				3.3	PRJNA281699
6	1.88	Transferase	LOC_Os06g04380	−1.7	PRJNA557063
				−1.7; −0.8	PRJNA281699
6	2.56	*OsNAPL2*	LOC_Os06g05660	2.1; 2.3	PRJNA448164
6	2.63	Transferase	LOC_Os06g05790	−2; −2.1	PRJDB2600
6	2.78	*OsFBL27*	LOC_Os06g06050	−3.5	PRJNA448164
				−1.9; −2.8	PRJNA430015
6	2.80	Esterase	LOC_Os06g06080	−2.7	PRJNA610422
				−2.0; −4.1	PRJNA448164
				−2.0	PRJNA354683
6	2.81	*OsMPK6*	LOC_Os06g06090	−1.4; −1.2	PRJNA448164
6	27.40	*OsFKBP13*	LOC_Os06g45340	−2.0; −7.2	PRJNA437981
				−4.6	PRJNA495106
6	27.78	*CYP59A*	LOC_Os06g45900	−1.8; −2.0; −3.0	PRJNA430015
6	28.04	*OsNAC011*	LOC_Os06g46270	−2.0; −2.4; −4.3; −3.3	PRJDB2600
6	28.05	Hydrolase	LOC_Os06g46284	1.3; 1.5	PRJNA610422
8	0.54	*RF6*	LOC_Os08g01870	−2.0; −2.1; −3.0; −3.2	PRJEB22031
8	4.65	*OsbHLH070*	LOC_Os08g08160	2.2; 2.9	PRJNA607661
8	16.86	*LTPL130*	LOC_Os08g27674	na	
8	17.56	*OsRLCK253*	LOC_Os08g28710	−3.7	PRJNA340947
				18.4	PRJNA430015
11	4.97	*OsPDIL1-1*	LOC_Os11g09280	1.5; 1.3	PRJNA437981
				3.9; 7.1	PRJNA448164
11	5.03	*OsFBX398*	LOC_Os11g09360	ns	

a Yu et al. (2022).

ns, not significant; na, no available expression data.

## Conclusions

In this study, we used two biparental mapping populations made from crosses between cold-tolerant and cold-sensitive parents (**Figure 1**) to fine-map cold tolerance QTL. We first performed “quality control” QTL analyses to demonstrate that heading date and plant height QTL contained known genes for those traits, such as *Ehd4*, *Ghd7*, *Hd1*, and *OsGA3ox4*, thus validating that the populations were suitable for cold tolerance trait QTL mapping (**Table 3**; **Supplementary Table S5**). All the 16 cold tolerance QTL identified here overlapped with the QTL we previously obtained through GWAS mapping approaches. This allowed us to narrow down the size of previously mapped QTL and identify 25 cold-tolerance candidate genes with mostly high-impact nucleotide variants between the cold-tolerant parents (Krasnodarskij 3352 and WIR 911) and the cold-sensitive parent (Carolino 164). Interestingly, most of those genes could be assigned to modules at the seedling stage that regulate osmolytes, ROS, and growth and development, as described in a recent rice cold tolerance review (Li et al., 2022). Specifically, five genes (20%) encoded receptor-like kinases that might be involved in the signal transduction of cold stress tolerance responses. That is, *OsMPK6*, *OsRLK-803*, *OsRLCK253*, *OsWAK38*, and *OsWAK40* might be part of modules regulating osmolyte and/or ROS production, together with the two (8%) transcription factor genes *OsbHLH070* and *OsNAC011*, and the two (8%) F-box genes *OsFBL27* and *OsFBX398* that might regulate transcription factor activities. The other 16 (64%) are potential cold stress tolerance effector genes that code for the following proteins: three (12%) chaperons that might help maintain biological macromolecule structure during cold stress; four (16%) ANK-TPR proteins that mediate interactions with protein partners of protein complexes with potential roles in cold stress tolerance responses; and nine (36%) enzymes involved in cellular metabolisms such as ROS production, maintenance of mitochondrial and/or chloroplast integrity, or functioning as ATPases, esterases, isomerases, hydrolases, or transferases. Of those, the two peptidyl-prolyl *cis*/*trans* isomerase genes *OsFKBP13* and *CYP59A* might belong to a new module regulating the trade-off between stress response and growth and development, as previously shown for *OsCYP20-2* (Ge et al., 2020). These candidate genes can be functionally analyzed in future studies using genomics, molecular genetics, and biochemical approaches, while the QTL regions containing those genes can be used for marker-assisted breeding of cold-tolerant rice cultivars.

## Data availability statement

The original contributions presented in the study are included in the article/**Supplementary Material**, further inquiries can be directed to the corresponding authors.

## Author contributions

MS: Conceptualization, Formal Analysis, Funding acquisition, Investigation, Methodology, Project administration, Supervision, Writing – original draft, Writing – review & editing. ARJ: Investigation, Writing – review & editing. AJ: Formal Analysis, Methodology, Software, Visualization, Writing – review & editing. HP: Investigation, Methodology, Writing – review & editing. MJ: Data curation, Methodology, Validation, Writing – review & editing. JE: Formal Analysis, Methodology, Software, Validation, Writing – review & editing. GE: Conceptualization, Funding acquisition, Investigation, Resources, Supervision, Validation, Writing – review & editing.
