# Supplementary material for: Navigating rice seedling cold resilience: QTL mapping in two inbred line populations and the search for genes
Source: Front Plant Sci. 2023 Dec 14;14:1303651. doi: 10.3389/fpls.2023.1303651 (PMC10755946; doi:10.3389/fpls.2023.1303651)
Supplement: Supplementary file 1 [file DataSheet_1.docx]

**Navigating Rice Seedling Cold Resilience: QTL Mapping in Two Inbred Line Populations and the Search for Genes**

*Michael R. Schläppi^1*^, Avery R. Jessel^1^, Aaron K. Jackson^2^, Huy Phan^1^, Melissa H. Jia^2^, Jeremy D. Edwards^2^, Georgia C. Eizenga^2*^*

^1^Department of Biological Sciences, Marquette University, Milwaukee, WI 53233, USA

^2^USDA-ARS Dale Bumpers National Rice Research Center, Stuttgart, AR 72160, USA

****Correspondence*:**

Michael Schläppi Georgia Eizenga

Department of Biological Sciences USDA-ARS

Marquette University DBNRRC

Milwaukee, WI 53233 Stuttgart, AR 72160

USA USA

Email: [michael.schlappi@marquette.edu](mailto:michael.schlappi@marquette.edu) [georgia.eizenga@usda.gov](mailto:georgia.eizenga@usda.gov)

Phone: +1 414 288-1480 Phone: +1 870 672-6104

**Keywords**: *aus* rice, chilling tolerance, heading date, IciMapping, *japonica* rice, plant height, RiceVarMap


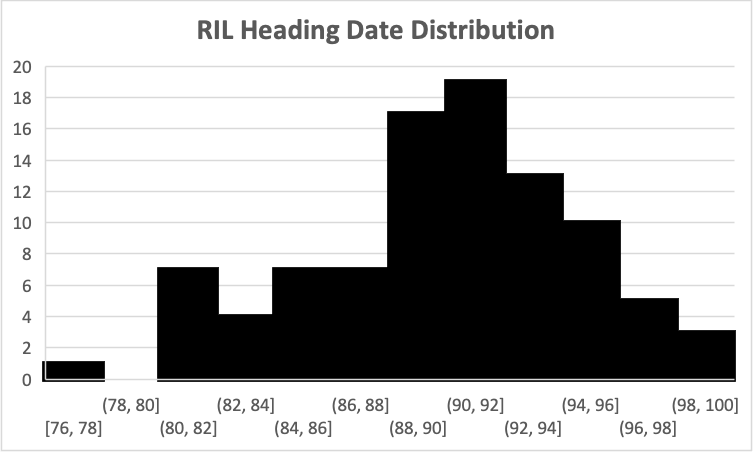

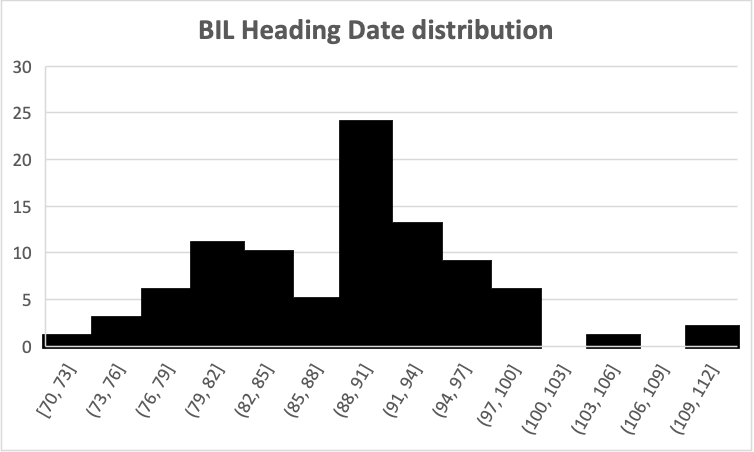


**Heading Date bins**

**Number of Plants**

**A**

Krasnodarskij 3352

WIR 911

Carolino 164

Carolino 164

**Supplementary FIGURE S1A.** Frequency distribution of heading date in F_8:9_ progeny of recombinant inbred lines derived from Krasnodarskij 3352 x Carolino 164 (RIL; left side), and in BC_1_F_5:6_ progeny of backcross recombinant inbred lines derived from [WIR 911 x Carolino 164] x Carolino 164 (BIL; right side). Arrows indicate the heading date values for the three parents.


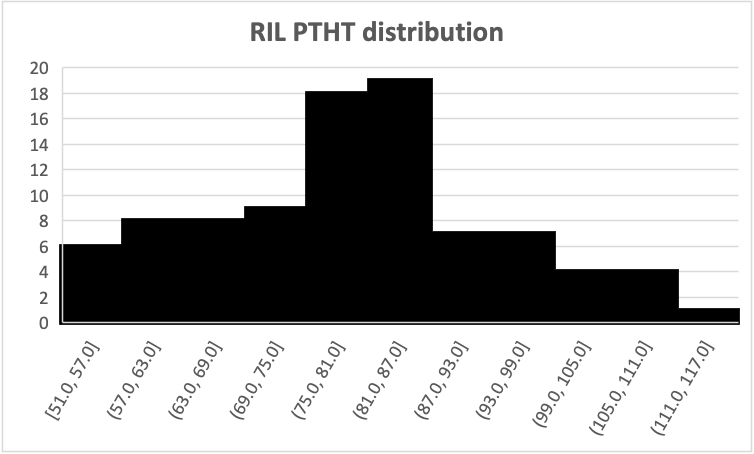

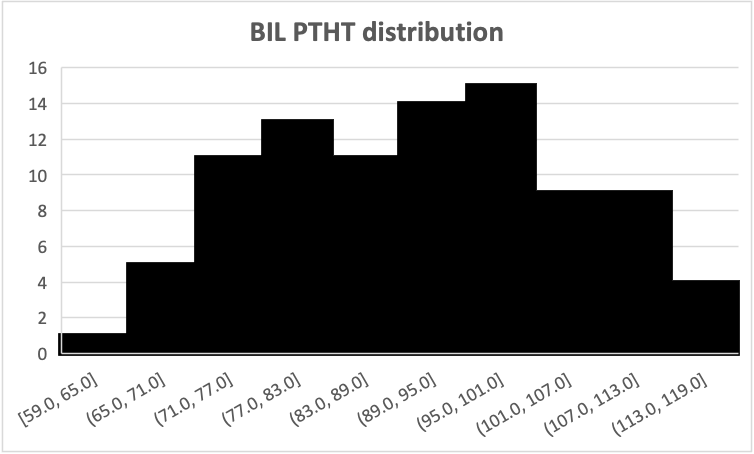


**Plant Height (PTHT) [cm] bins**

**Number of Plants**

**B**

Krasnodarskij 3352

WIR 911

Carolino 164

Carolino 164

**Supplementary FIGURE S1B.** Frequency distribution of mature plant height (PTHT, measured in cm from soil level to tip of mature panicle) F_8:9_ progeny of recombinant inbred lines derived from Krasnodarskij 3352 x Carolino 164 (RIL; left side), and in BC_1_F_5:6_ progeny of backcross recombinant inbred lines derived from [WIR 911 x Carolino 164] x Carolino 164 (BIL; right side). Arrows indicate the PTHT values for the three parents.


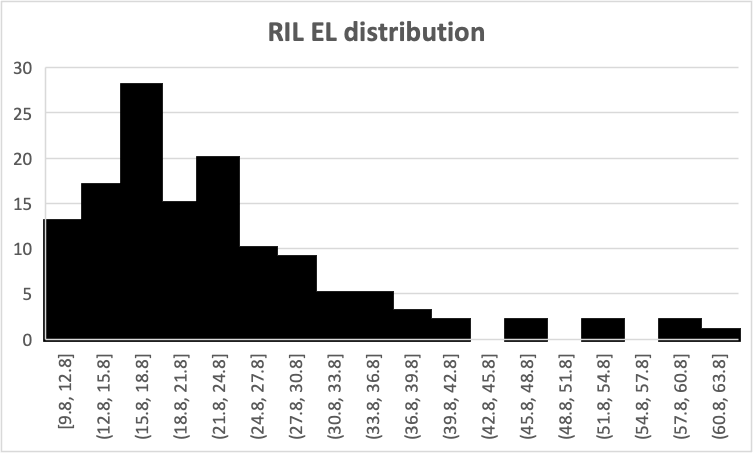

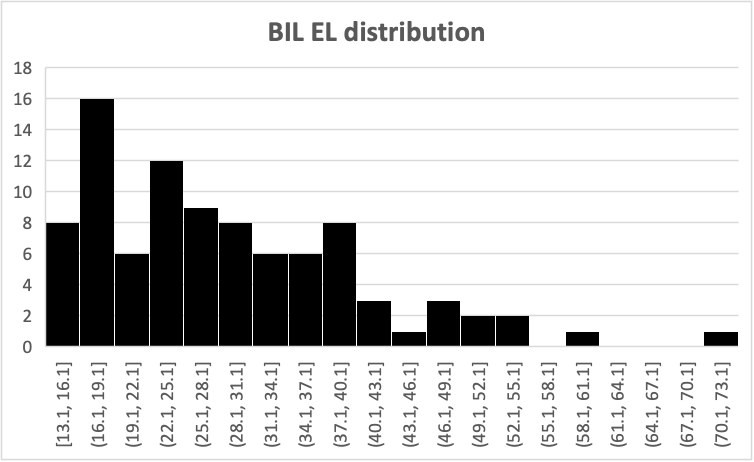


**Percent Electrolyte Leakage (EL) bins**

**Number of Plants**

**C**

Krasnodarskij 3352

WIR 911

Carolino 164

Carolino 164

**Supplementary FIGURE S1C.** Frequency distribution of percent electrolyte leakage (EL) in leaves of F_8:9_ progeny of recombinant inbred lines derived from Krasnodarskij 3352 x Carolino 164 (RIL; left side), and in BC_1_F_5:6_ progeny of backcross recombinant inbred lines derived from [WIR 911 x Carolino 164] x Carolino 164 (BIL; right side) after a one-week exposure to constant 10°C. Arrows indicate the EL values for the three parents.


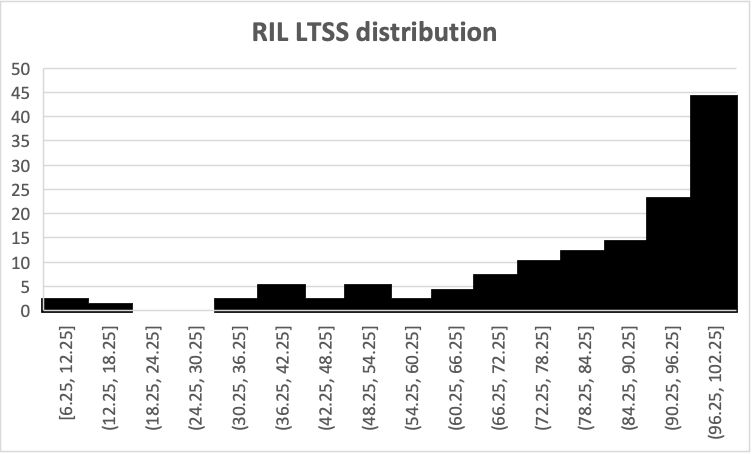

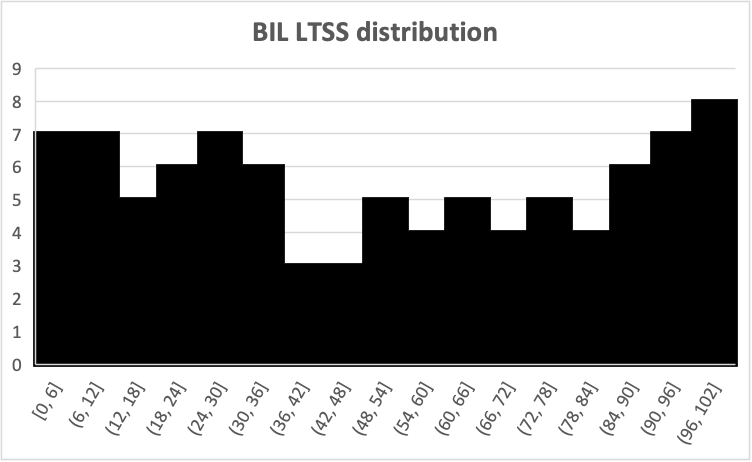


**Percent Low-Temperature Seedling Survivability (LTSS) bins**

**Number of Plants**

**D**

Krasnodarskij 3352

WIR 911

Carolino 164

Carolino 164

**Supplementary FIGURE S1D.** Frequency distribution of percent low-temperature seedling survivability (LTSS) of F_8:9_ progeny of recombinant inbred lines derived from Krasnodarskij 3352 x Carolino 164 (RIL; left side), and in BC_1_F_5:6_ progeny of backcross recombinant inbred lines derived from [WIR 911 x Carolino 164] x Carolino 164 (BIL; right side) after a one-week exposure to constant 10°C. Arrows indicate the LTSS values for the three parents.
